# Association between local spatial accessibility of dental care services and dental care quality

**DOI:** 10.1186/s12903-021-01943-z

**Published:** 2021-11-17

**Authors:** Ping-Chen Chung, Ta-Chien Chan

**Affiliations:** 1 Department of Dentistry, Puzi Hospital, Ministry of Health and Welfare, Chiayi, Taiwan; 2 Research Center for Humanities and Social Sciences, Academia Sinica, 128 Academia Road, Section 2, Taipei, Taiwan; 3 Institute of Public Health, School of Medicine, National Yang Ming Chiao Tung University, Taipei, Taiwan

**Keywords:** Accessibility, Two-step floating catchment area (*2SFCA*), Dental care quality

## Abstract

**Background:**

The aim of our study was to evaluate the allocation of dental resources and explore access to dental care in Taiwan. In addition, we tried to understand the spatiotemporal characteristics of dental care quality and analyze the relationship between dental care quality and areas with deficiencies in dental resources.

**Methods:**

The study used a two-step floating catchment area to calculate the dental resources accessibility and explore the spatiotemporal distributions of dental care quality. The association between dental care quality and spatial accessibility was analyzed using a spatial error model.

**Results:**

Most areas with deficient dental resources and lower dental care quality were remote townships, agricultural towns, or aging towns with spatial clustering. The quality of children's preventive dental care had increased over time. Most highly urbanized areas had higher dental care quality. The quality of some dental care types such as children's preventive care and full-mouth calculous removal was associated with higher accessibility.

**Conclusions:**

Understanding the spatiotemporal distribution of both dental care accessibility and quality can assist in allocation of dental care resources. Adequate dental resources may elevate dental care quality. Suggestions include policies to balance dental resources and routinely monitor improvement in areas with deficient dental care.

**Supplementary Information:**

The online version contains supplementary material available at 10.1186/s12903-021-01943-z.

## Background

Oral care is an important public health issue. In most countries, especially in developing countries, there are not enough dental resources. Even in developed countries, the dental-to-population ratios have inequalities in different regions, and this is especially a concern among deprived communities, certain ethnic minorities, and disabled individuals [1]. For example, the density of dentists in 2019 based on American Dental Association Health Policy Institute data varied by state. The highest density was 104 dentists per 100,000 population in the district of Columbia, and the lowest was 40.97 in Alabama [2]. Heterogeneity of dental resources has been widely found, with differences between rural and urban areas and across countries.

Similarly, Taiwan’s medical resources are distributed unevenly; most are in high-population-density and urban areas and less are in remote areas such as mountainous and hilly regions, which account for over two-thirds of the nation’s land area [3]. Inconvenient traffic transportation, inadequate medical equipment and insufficient medical personnel in remote areas cause low medical accessibility [4, 5]. In the eastern region of Taiwan, Hualien city, located on flatlands, is the most densely populated area in Hualien county, and has more medical resources than other townships. According to 2018 Hualien county statistics, the ratio of dentists per 10,000 population was 9.91 in Hualien city and only 0.62 in Xiulin township, a mountain indigenous township [6]. The report also showed that most medical resources are concentrated on the plains, and there is a lack of resources in mountainous townships and coastal areas.

There are several factors affecting dentists’ choice of location for their practice and causing uneven distribution of dental resources. First, variation in both the awareness of oral health and knowledge of the importance of dental care cause different degrees of demand for dental services. Second, the ability to pay for services is different, especially for the poor, minorities, uninsured people and people with relatively poor health [7, 8]. Third, if dentists practice in remote areas, it may increase their workload and difficulty in recruiting staff [9, 10].

Uneven distribution of dental resources affects the timing of receiving dental treatment and transportation time to dental facilities. For example, people in remote areas of Hualien county spend an average of 16 min to arrive at the nearest medical institution (standard error: 33.3 min), and people in the villages of Hualien county spend an average of 7 min (standard error: 7.6 min) [5]. More people in remote areas expressed that seeking medical service was inconvenient [5]. In a study exploring the effects of distance to dentists on children’s dental service usage, increased distance of 3.3 miles reduced having comprehensive oral exams by about 6% [11]. Moreover, unevenness of medical resources influences residents’ health status and induces health disparities. In remote areas, it is difficult to accumulate health capital due to resource accessibility, transportation costs and travel time [12, 13].

Many medical accessibility definitions have been proposed, among which Penchansky and Thomas [14] divided accessibility into five domains, including availability, accessibility, accommodation, acceptability and affordability. The first two are related to spatial factors, which are the type and quantity of medical resources within a specific space and the convenience of transportation including transportation time, distance and cost. Others are related to demographic characteristics, healthcare needs, socioeconomic status and cultural differences. Many methods for measuring potential resource accessibility have been used, including calculating the nearest or average distance, dentist-to-population ratio, and the two-step floating catchment area (2SFCA) method [15, 16]. Luo and Wang proposed the 2SFCA method in 2003 and used two catchments to evaluate resource usage [16]. This method deals with the limitation of searching for medical resources only within administrative boundaries, because people may seek medical services across boundaries within a manageable distance.

Previous studies have explored dental resources distribution by dentist-to-population ratio [17]. To our knowledge, few studies have applied the 2SFCA method to analyze access to dentists. Moreover, caries prevention [18, 19], periodontal care [18] and tooth restoration longevity [20] may be used as markers of quality in dentistry. No prior research has explored whether dental care quality differs between areas with adequate versus deficient dental resources. The aim of this study was to use 2SFCA to explore dental resource accessibility, the spatiotemporal distribution of dental care quality, and the association between resource-deficient areas and dental care quality [21].

## Methods

### Data resources

Mid-year population data at the village level from 2012 to 2019 were downloaded from socio-economic databases maintained by the Ministry of the Interior, Taiwan (https://segis.moi.gov.tw/STAT/Web/Portal/STAT_PortalHome.aspx). Medical institutions and personnel statistical data obtained from an open data platform in Taiwan (https://data.gov.tw/) were used to geocode the location of the dentists’ practice addresses. A caries experience index (decayed, missing, and filled teeth, DMFT index) was obtained from an investigation on the oral hygiene and status of children and adolescents six to 18 years old in Taiwan between 2009 and 2011 [22]. The index was used as an indicator of oral status in each county. The caries experience index is the sum of the total number of caries, teeth extracted due to caries, and teeth filled.

Three quality indexes including dental filling preservation rate (within 2 years), calculus removal rate (13 years old or older) and fluoride service rate (under 6 years old) were captured from the National Health Insurance open data platform (https://data.nhi.gov.tw/Index.aspx) between 2012 and 2018 and the first to third quarters in 2019. According to an official quality index definition and value for inference [23], the dental filling preservation rate (within 2 years) is the proportion of not repeating filling of the same tooth in the same dental facility in 2 years for a specific period. When dental fillings remain intact for more than 2 years, it is likely to be partly due to the choice of filling materials and correct and skilled operation [23]. The calculus removal rate (13 years old or older) is the proportion of 13-year-old or older patients receiving full mouth cleaning with ultrasonic scaling for a specific period. The calculus removal rate reflects the dentists performing periodontal disease care and regular full-mouth scaling [23].

The fluoride service rate (under 6 years old) is the proportion of performing oral preventive and care services for a specific period. Children receive dental facility or community tour service once every 6 months by using national health insurance. Children from low-income households, indigenous and remote areas or those with physical and mental disabilities can receive fluoride service every 3 months. The fluoride service rate reflects parents’ attitude and cognition, children’s cooperation, and willingness of the dentist practice. Fluoride service can prevent primary tooth decay and make up for unskilled tooth brushing by children [23].

The urbanization degree of 359 townships in Taiwan is classified into seven types, including highly urban, moderately urban, emerging, general, aging, agriculture town and remote township, as proposed by Liu et al. [24].

### Travel time to the closest provider

In previous studies, the average weighted medical service distance was 10 km in highly urban, moderately urban and emerging towns in Taiwan [25]. Overall, the average weighted medical service distance was 17.68 km. Considering that the average road speed limit is 60 km/h [26], we set 10 min as the threshold to evaluate the spatial accessibility.

### Dental resource accessibility analysis: two-step floating catchment area

In the first step, we set a 10-min catchment area around each dental facility using OpenStreetMap [27] as a base map, identified all the population within each catchment, and calculated supply-to-demand ratios for each dental facility with QGIS 3.4.7 [28] and ORS Tools (Version 1.2.3) [29]. In the second step, we summed supply-to-demand ratios in each catchment around the geographical centroid of each village. The sum of supply-to-demand ratios for each village was its accessibility score. The data sources mentioned above are listed in the Additional file 1.

### Provider to population ratios

It has been suggested that one dentist serves 2000 people per year, and areas with one dentist for 4000 people or more per year are defined as dental-resource-deficient areas [30]. After converting the above dentist service quantity to spatial accessibility, we see that a person can make 0.0005 dentist service trips and 0.00025 dentist service trips per year in normal and dental-resource-deficient areas respectively.

### Statistical analysis

We visualized the dental resources accessibility at the village level and three dental care quality indexes at the township level, and defined deficient townships based on two accessibility scores, 0.0005 and 0.00025. The temporal trends of the three dental care quality indexes were plotted by box plot and also stratified by the urbanization degree at the township level. Local indicators of spatial association (LISA) were applied to measure spatial dependence and evaluate localized spatial clusters of dental resources and three dental care quality indexes using a QGIS Spatial Autocorrelation Map [31]. Because of adjacency spatial dependency, a spatial error model with rook contiguity was used to explore the association between dental resource accessibility and dental care quality after adjusting for the DMFT index at the county level in 2012. All analyses used QGIS 3.4.7, GeoDa (subversion 1.14.0), SAS (version 9.4) and RStudio (Version 1.0.153), and all statistical tests were two-sided with a significance level of 0.05.

## Results

**Fig. 1:**
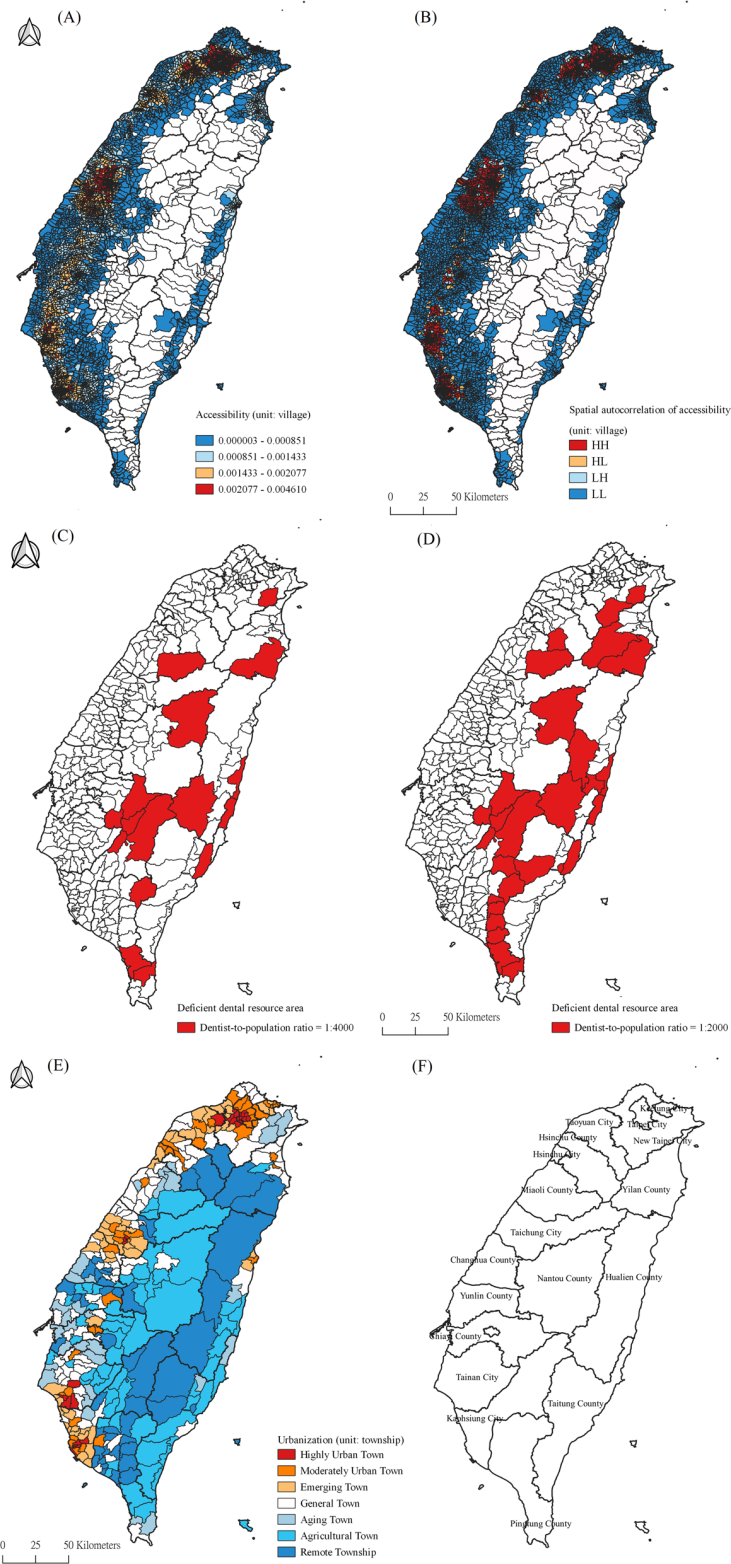
Spatial distribution of dental resources and urbanization. **a** Accessibility of dental resources (unit: village). **b** LISA of accessibility (unit: village). **c** Dental-resource-deficient area (dentist-patient ratio = 1:4000; unit: township). **d** Dental-resource-deficient area (dentist-patient ratio = 1:2000; unit: township). **e** Urbanization degree (unit: township). **f** List of county names. Note: The figure is created by this paper’s authors

This study involved 7604 villages on the main island of Taiwan. The villages located on the western coast, and in eastern Taiwan and the mountainous areas had low accessibility, shown in blue (Fig. 1a). The red areas indicate the places with sufficient resources, most of which were concentrated in metropolitan areas such as Taipei City, New Taipei City, Taichung City, Tainan City and Kaohsiung City. Corresponding to the level of urbanization of each township, resources were concentrated in the west, especially in the western metropolitan areas, and the areas with low accessibility of dental service were mostly in aging, agricultural and remote towns (Table 1). The ratios of the population to the dentists were also inversely correlated with the spatial accessibility. LISA found spatial clusters, which revealed hot spots (high–high), defined as areas with a lot of dental resources whose neighbors have the same phenomenon. These hot spots were concentrated in the western metropolitan areas, and the cold spots were scattered around the western coast, the east and the surrounding mountains (Fig. 1a).

**Table 1:** Descriptive statistics of spatial accessibility of dentist resources according to the degree of urbanization

Degree of urbanization	Number of villages	Total population	%	Average P/D	SE P/D	Average R	SD R
Highly Urban Town	1267	5,332,102	22.85	1286.20	189.88	0.02837	0.00857
Moderately Urban Town	1709	7,247,121	31.06	1577.81	97.79	0.01771	0.00733
Emerging Town	1260	4,978,888	21.34	3693.84	334.97	0.01679	0.00676
General Town	1587	3,447,247	14.77	6542.71	582.35	0.01084	0.00597
Aging Town	431	442,748	1.90	11,552.03	1725.02	0.00630	0.00390
Agricultural Town	739	925,244	3.97	8623.77	778.95	0.00561	0.00467
Remote Township	611	960,034	4.11	9036.32	957.44	0.00721	0.00518
Total	7604	23,333,384	100.00	5820.99	315.10	0.01523	0.00989

P/D, The ratio of the population to the dentists; R, Spatial accessibility

The thresholds of accessibility scores were 0.0005 and 0.00025 (Fig. 1b) at the township level, respectively. In other words, the dentist-to-population ratios were 1:2000 and 1:4000, and the former cutoff point covered more deficient areas. In the 352 towns, dental-resource-deficient areas accounted for 4.5% (n = 16) under the 0.00025 threshold and were concentrated in the mountainous areas and the east.

Villages were divided into four groups by quartiles, with a fifth group being those completely lacking dental resources (Table 2). 267 villages had no dentist within 10 min’ driving distance and contained 250,906 persons (accounting for 1.08% of the total population). 1834 villages with low accessibility (0 < R ≦ 0.00851) included 3,006,980 people (accounting for 12.89% of the total population). The areas with the second lowest accessibility (0.00851 < R ≦ 0.01433) included 1840 villages, with 4,469,234 people (19.15% of the total population). The slightly higher accessibility (0.01433 < R ≦ 0.02077) covered 1831 villages and included 6,849,317 people (accounting for 29.35% of the total population). In addition, there were 1832 villages with high accessibility (0.02077 < R) including population of 8,756,947 (maximum percentage of total population: 37.53%).

**Table 2:** Descriptive statistics of spatial accessibility of dentist resources in each village

Spatial accessibility	Number of villages	Total population	%	Average R	SD R
R = 0	267	250,906	1.08	0	0
0 < R ≦ 0.00851	1834	3,006,980	12.89	0.00513	0.00223
0.00851 < R ≦ 0.01433	1840	4,469,234	19.15	0.01129	0.00163
0.01433 < R ≦ 0.02077	1831	6,849,317	29.35	0.01741	0.00177
0.02077 < R	1832	8,756,947	37.53	0.02935	0.00625
Total	7604	23,333,384	100.00	0.01523	0.00989

R: Spatial accessibility

**Fig. 2:**
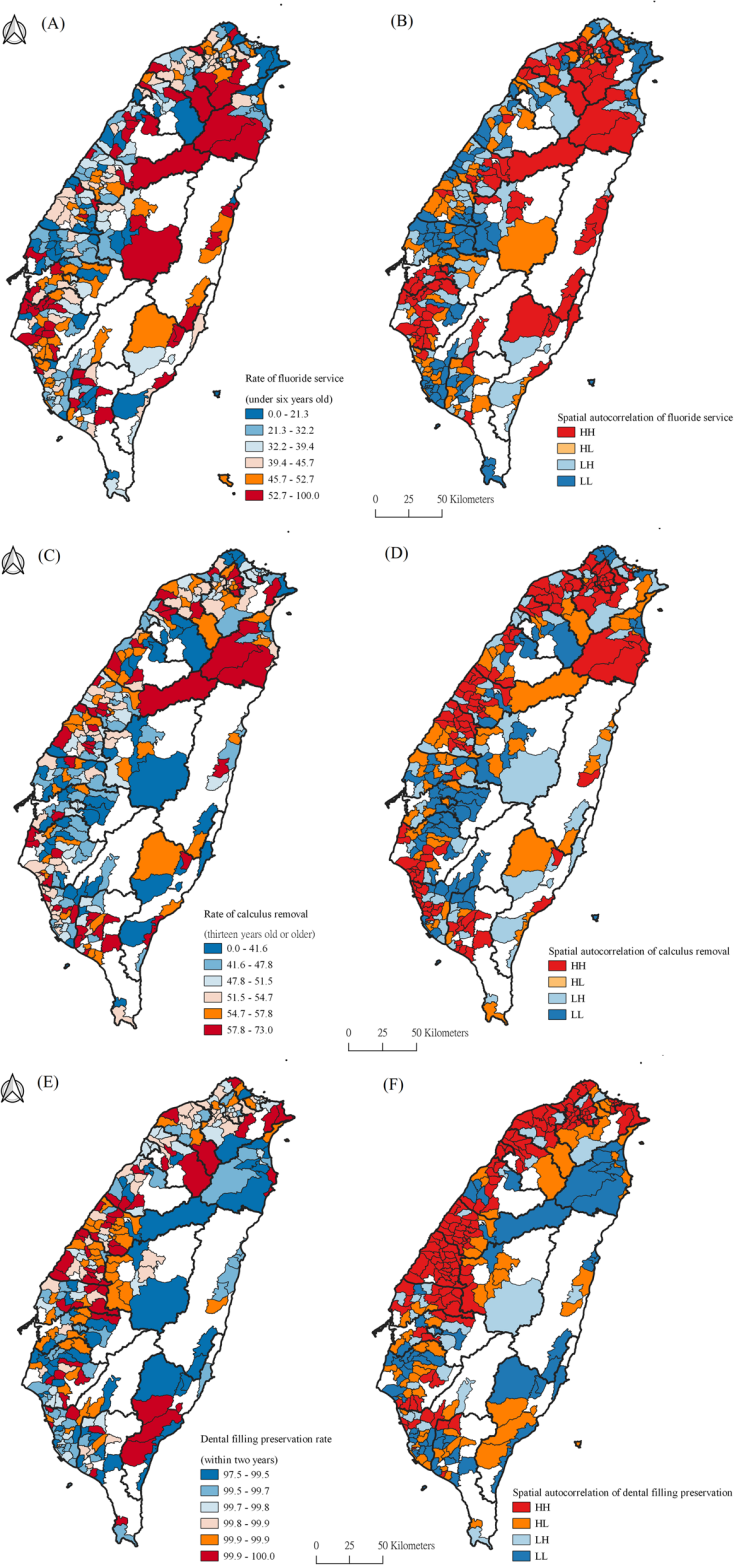
Spatial distribution of dental quality indexes (unit: township). **a** Percentage of children with fluoride service (under 6 years old). **b** LISA of children with fluoride service (under 6 years old). **c** Percentage of patients with calculus removal (13 years old or older). **d** LISA of patients with calculus removal (13 years old or older). **e** Dental filling preservation rate (within 2 years). **f** LISA of dental filling preservation rate (within 2 years). Note: The figure is created by this paper’s authors

Low proportions of fluoride service (for children under 6 years old) were concentrated on the northeast coast and sporadic areas of the central western plains, especially in areas where the urbanization levels were aging, agricultural and remote townships. The hot zones of spatial clusters were concentrated in the north, southwest and east, while the cold zones were in the northeast coast, and on the western plain with its ageing, agricultural and remote townships, and the southern coast (Fig. 2a).

The proportion of calculus removal (13-year-olds or older) was higher on the sporadic western plains and in the east, and lower in the sporadic middle part of the west, southeast and mountainous areas. The hot zones were in the northeast and western plains, especially in the metropolitan areas, and the cold zones were concentrated in the middle and southern parts of mountainous areas and the northern coast (Fig. 2b).

The proportion of dental filling preservation (within 2 years) accounted for more than 95% of filled teeth in all counties. The places with lower refilling rates were in the north and central parts of Taiwan. The hot zones of spatial autocorrelation distribution were in the north and middle parts of the west, and the cold zones were in the east and the sporadic regions of the south (Fig. 2c).

**Fig. 3:**
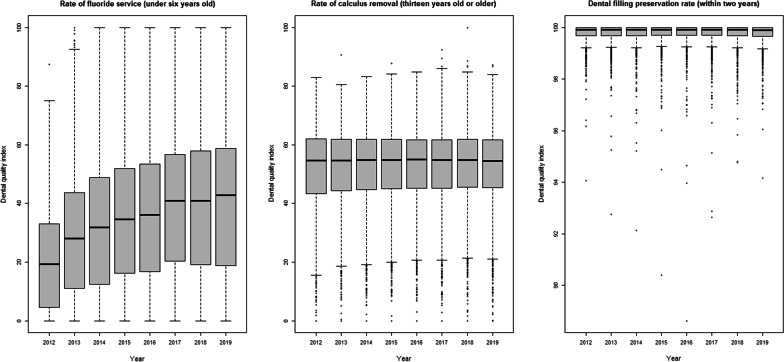
Dental quality indexes in Taiwan from 2012 to 2019

**Fig. 4:**
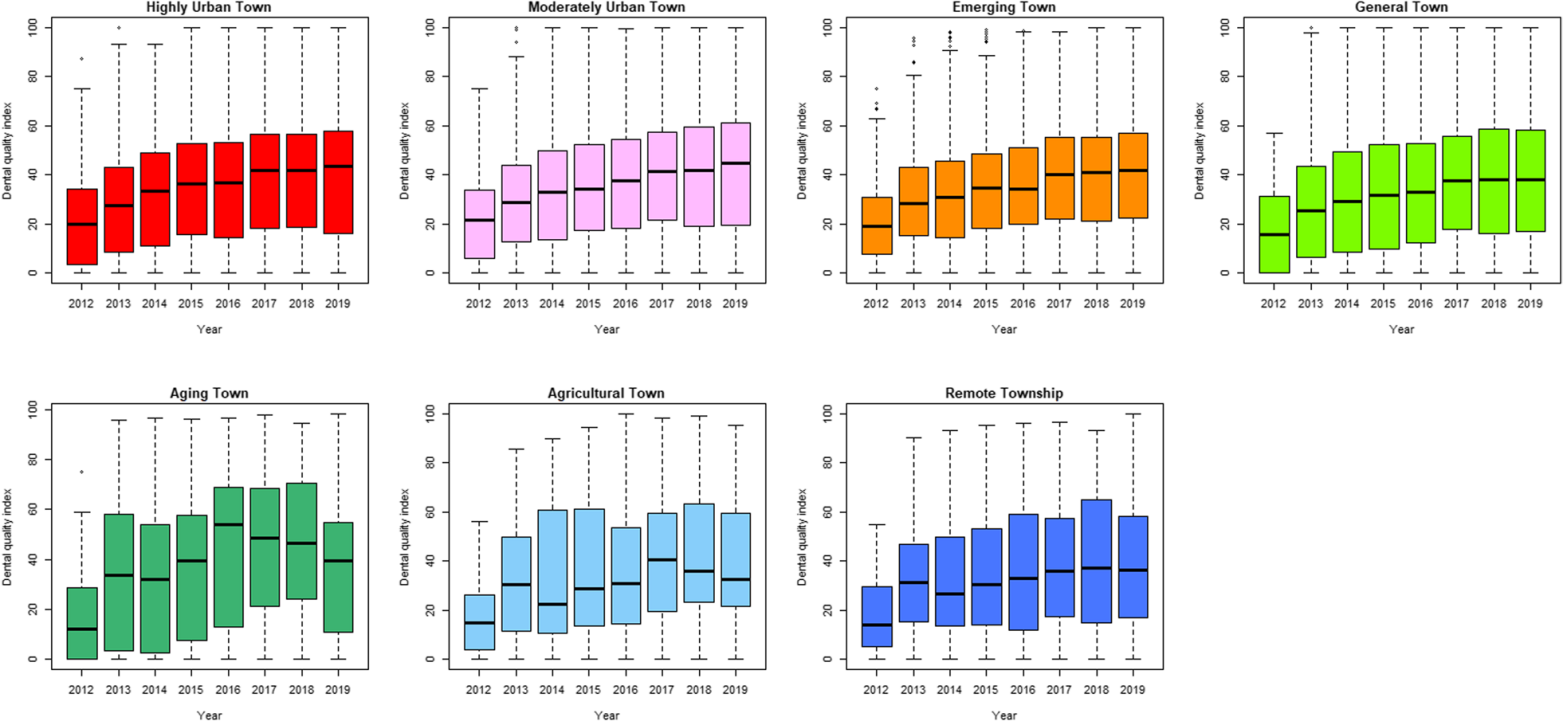
Rate of fluoride service (under 6 years old) in different degrees of urbanization from 2012 to 2019

**Fig. 5:**
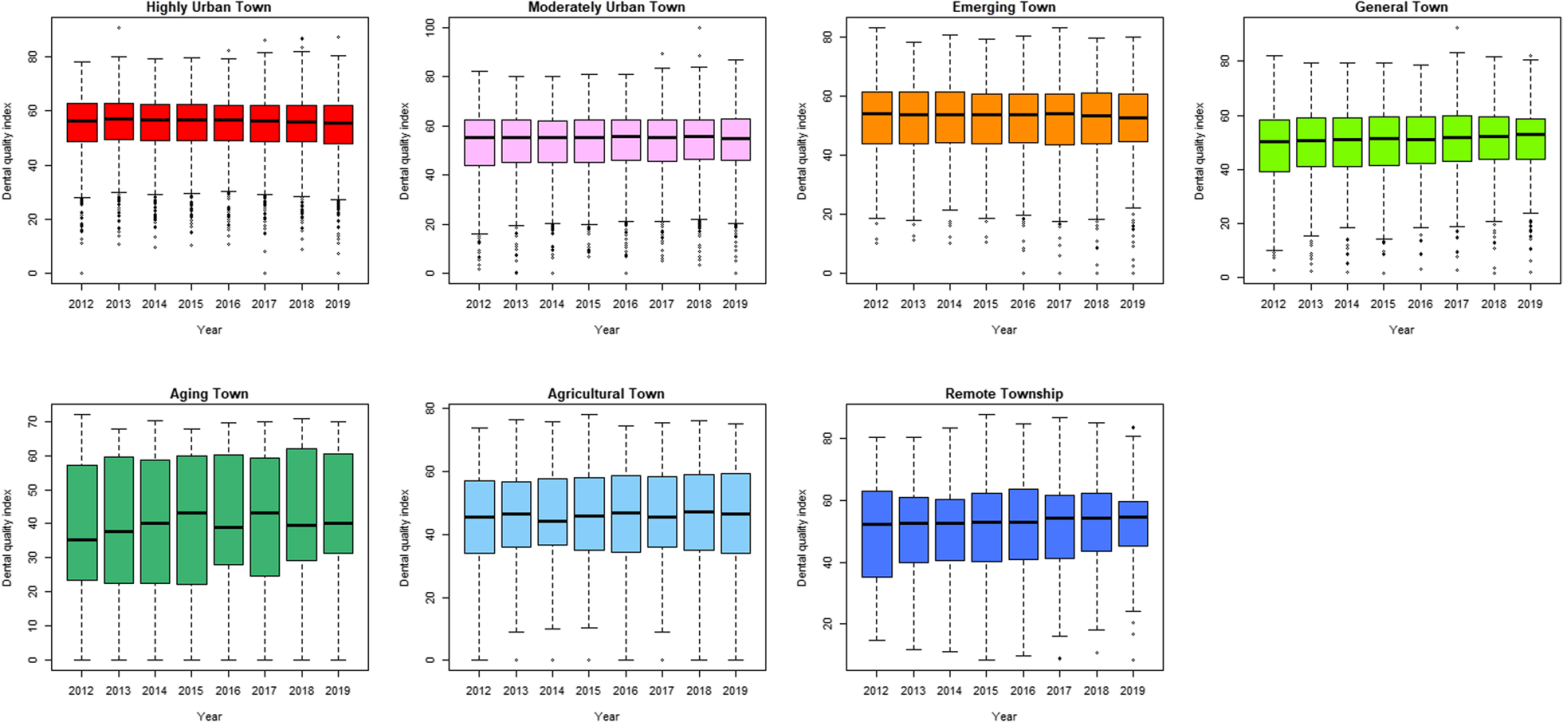
Rate of calculus removal (13 years old or older) in different degrees of urbanization from 2012 to 2019

**Fig. 6:**
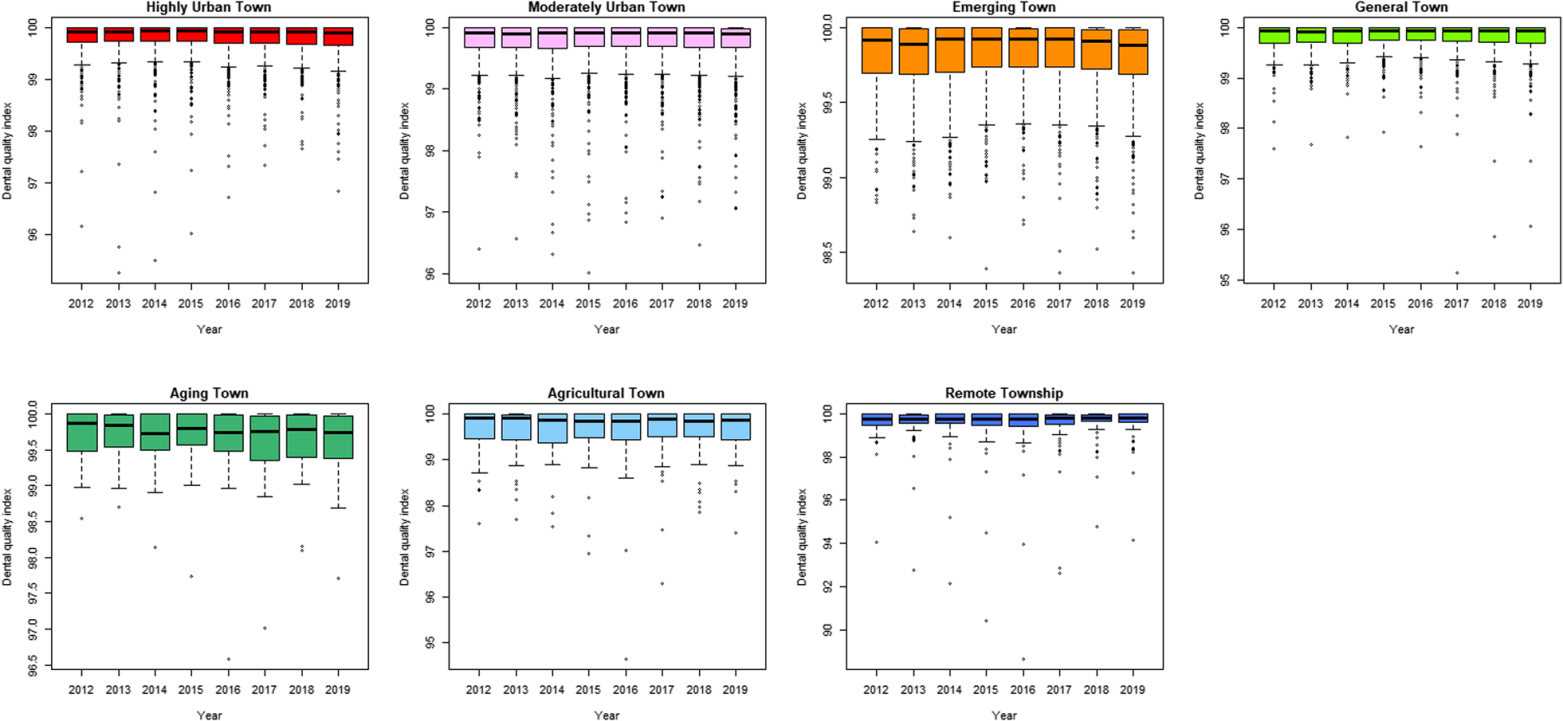
Dental filling preservation rate (within 2 years) in different degrees of urbanization from 2012 to 2019

Performing fluoride service (for children under 6 years old) increased year by year from 2012 to 2019, and the median value in 2019 was 42.86% (interquartile range, IQR = 39.91). The rate of calculus removal (13 years old or older) and dental filling preservation rate (within 2 years) had little difference over time. The former had a median index of 54.52% in 2019 (IQR = 16.25%), and the latter had a median index of 99.90% in 2019 (IQR = 0.33%) (Fig. 3). Fluoride service (under 6 years old) increased year by year from 2012 to 2019 in highly urban, moderately urban, emerging and general towns. But in aging, agricultural and remote townships the fluoride implementation rate fluctuated with time (Fig. 4). The rate of calculus removal (13 years old or older) and dental filling preservation rate (within 2 years) had no particular change over time for different urbanization levels, except for the aging towns, which showed a slight fluctuation (Figs. 5, 6). The rates of fluoride service (under 6 years old) and calculus removal (13 years old or older) in general, aging, agricultural and remote townships were lower than those in highly urban, moderately urban and emerging towns. The dental filling preservation rate (within 2 years) was more than 95% across different urbanization levels, and the difference in each urbanization level was small.

In the spatial error model, regarding the fluoride service (for those under 6 years old), the dental service accessibility (coefficient = 7.17E−05, *p* value = 0.002) and the caries experience index (coefficient = −3.73, *p* value ≤ 0.0001) were significant. The model interpretation power (R-square) was 0.665, with spatial dependence (*p* value = 0.023). Regarding calculus removal rate, the dental service accessibility (coefficient = 1.10E−04, *p* value = 0.004) was significant, and the model's explanatory power was 0.345, but there was no spatial dependence (*p* value = 0.158). Regarding the dental filling preservation rate (within 2 years), the dental service accessibility (coefficient = −3.01E−07, *p* value = 0.679) and the caries experience index (coefficient = −0.0008, *p* value = 0.972) were not significant, but there was spatial dependence (*p* value = 0.045). The model's explanatory power was 0.278 (Table 3).

**Table 3:** Spatial error model of different quality indexes in 2012 (spatial unit: county or city)

Variable	Rate of fluoride service (under 6 years old)			Rate of calculus removal (13 years old or older)			Dental filling preservation rate (within 2 years)		
	Coeff	SE	*p* value	Coeff	SE	*p* value	Coeff	SE	*p* value
Accessibility (median)	7.17E−05	2.27E−05	0.002	1.10E−04	3.78E−05	0.004	− 3.01E−07	7.28E−07	0.679
Caries experience index (DMFT index)	− 3.73	0.79	< 0.0001	− 0.82	1.30	0.527	− 0.0008	0.02	0.972

Diagnostics for spatial dependence	*p* value	*p* value	*p* value
Likelihood ratio test	0.023	0.158	0.045
*Model assessment*			
R-squared	0.665	0.345	0.278

Coeff = coefficient; Std. Error = Standard Error

## Discussion

In the traditional approach, the dentist-to-population ratio was calculated by fixed administrative boundaries and couldn’t consider spatial heterogeneity. Our study used the road network and 2SFCA to compute the accessibility of dental resources while overcoming the difficulty of cross-boundary accessibility estimation. In addition, the usage of dental facility addresses was more accurate to reflect the supply of dental resources. For example, medical services are more likely to concentrate in the busy section of district, and the dentist-to-population ratio can’t represent this feature. We used spatial autocorrelation analysis to reveal clusters, helping us understand dental resource distribution at the village level. The results can help government to target insufficient resources areas, provide corresponding dental resources, effectively establish and integrate a reasonable system, and reduce health inequality [15, 32, 33].

Lower accessibility of dental resources and a lower dental care quality index were mostly associated with remote, agricultural or aging towns with spatial clustering. Even distribution of the dental resources can elevate the usage of dental service in remote areas. In a cross-sectional study, which used Washington State Medicaid Program data in 2012, the proportion of Medicaid-enrolled children who utilized preventive dental care significantly increased by 1.67 percent as the ratio of pediatric dentists per 10,000 children in a county increased, after adjusting both for regionality and for the age of access to a baby and child dental program which was a special Medicaid access program to improve the oral health of those under age 6 years [34]. One study which enrolled children between 2000 and 2009 from Iowa Medicaid found that increased distance to the nearest dentist was associated with a decrease in comprehensive exams [11]. Generally speaking, urban areas are more likely to have sufficient resources and high-quality service. In one study identifying the dentally underserved geographic areas in the US, dentally underserved areas had significantly lower population densities regardless of urbanization level [35].

Dental care quality indexes have mostly risen or remained steady over time, and the indexes were relatively high in highly urban areas. There were some dental care quality indexes associated with accessibility such as rates of fluoride service (for those under 6 years old) and calculus removal (13 years old or older). The dental care quality as evaluated by a standard criterion allowed observation of the historical trends, comparison with other institutions in different regions, and an understanding of which types of quality needed to be improved. In this study, dental care quality indexes were calculated by health insurance claims data to represent the implementation of different treatments and preventive care in the different dental facilities. The health insurance claims data had expert review on medical care quality and expenses by the National Health Insurance Administration. There also exist other methods for measuring dental care quality. The Dental Quality Alliance, established by the American Dental Association, has developed standard and verifiable measurement [18], including oral care usage, oral care quality and cost to enhance oral health. Righolt et al. established a definition for quality of oral healthcare which comprises seven domains—patient safety, effectiveness, efficiency, patient centeredness, equitability, timeliness, and access to care [36]. This quality measurement, with its more comprehensive definition, can be used for routine feedback of information on the quality and outcome of oral health care, and to promote quality improvement in oral health care [37].

Preventive care of fluoride application for children under 6 years old should be performed once every 6 months. In remote areas where the population density is lower than one-fifth of the average population density, fluoride services can be conducted twice every 6 months. Fluoride community tour services are often performed in remote areas. For example, National Yang-Ming University Hospital in Taiwan regularly goes to remote areas in Nan'ao or Datong Township in Yilan County in northeastern Taiwan to offer fluoride application and caries prevention. Therefore, for these reasons, some remote areas have a high percentage of implementing preventive services.

There are several methods to improve accessibility in dental-resource-deficient areas. First, financial incentives of low interest rates for loan repayment or tax breaks can be used for attracting dental facilities located in dental-resource-deficient areas. Improving quality of life to attract dentists to practice in rural areas may also help balance the urban–rural skew. Second, increasing the demand by educating the underserved population on the need to maintain oral health attracts dentists to provide care in underserved areas [35]. Due to varying dentist working hours and specific services provided, the establishment of transparent service information may help residents search for the appropriate dental service. Third, implementing universal enrollment health insurance can help balance the geographic distribution of health providers, as confirmed in a previous study, in which implementing National Health Insurance improved the equality of dentist geographic distribution after controlling for the natural growth by the time trend in Taiwan [38]. Moreover, cost-effective mobile and portable dental services can be made available in resource-scarce or naturally isolated areas to solve the disparity in accessibility [39].

In terms of the research limitations of this study, first, the actual dentist practice time such as full time and part time was not considered. If a dental facility is closed when a patient needs treatment, the accessibility is reduced. Second, if a dentist is a specialist such as an orthodontist, she or he may not perform other treatment, and the accessibility of basic care will be overestimated. Third, the dental care quality index reflects the preventive and curative treatment performance, and other domains of dental care quality are not included in the national health insurance claims data. Furthermore, the dental filling preservation rate (within 2 years) cannot reflect a situation in which the same person has the same tooth filled in different dental facilities within 2 years, so the quality index value may be slightly higher.

## Conclusion

In conclusion, regular monitoring of dental services accessibility can help policymakers and health services providers reallocate dental resources and balance resource distribution by encouraging dentists to practice in remote areas and to provide mobile dental health care.

## Supplementary Information

**Additional file 1:** The summary of variables collected for dental resource accessibility analysis.

## Data Availability

The datasets analysed during the current study are available in the figshare repository [10.6084/m9.figshare.16907098].

## Abbreviation

2SFCA: Two-step floating catchment area
