# Supplementary material for: Association between local spatial accessibility of dental care services and dental care quality
Source: BMC Oral Health. 2021 Nov 17;21:582. doi: 10.1186/s12903-021-01943-z (PMC8600821; doi:10.1186/s12903-021-01943-z)
Supplement: Supplementary file 1 — Additional file 1: The summary of variables collected for dental resource accessibility analysis. [file 12903_2021_1943_MOESM1_ESM.docx]

Supplementary Table. The summary of variables collected for dental resource accessibility analysis

| Variable | Source/Software | Definition | Collected time points |
| --- | --- | --- | --- |
| Population | Socio-economic geographic information system | Mid-year population at village level | 2012 to 2019 |
| Statistical data on medical institutions and personnel | Ministry of the Interior open data platform | Dentist number and practice location | 2012 to 2019 |
| 10-minute catchment area | OpenStreetMap | Area reachable within 10 minutes of driving from each dental facility |  |
| Supply-to-demand ratios | QGIS 3.4.7and ORS Tools (Version 1.2.3) | Ratio of population to dentists |  |
